# Genetic basis for nitrate resistance in *Desulfovibrio* strains

**DOI:** 10.3389/fmicb.2014.00153

**Published:** 2014-04-21

**Authors:** Hannah L. Korte, Samuel R. Fels, Geoff A. Christensen, Morgan N. Price, Jennifer V. Kuehl, Grant M. Zane, Adam M. Deutschbauer, Adam P. Arkin, Judy D. Wall

**Affiliations:** ^1^Department of Biochemistry, University of MissouriColumbia, MO, USA; ^2^Ecosystems and Networks Integrated with Genes and Molecular AssembliesBerkeley, CA, USA; ^3^Department of Molecular Microbiology and Immunology, University of MissouriColumbia, MO, USA; ^4^Physical Biosciences Division, Lawrence Berkeley National LaboratoryBerkeley, CA, USA

**Keywords:** sulfate-reducing bacteria, sulfide control, *Desulfovibrio*, nitrite, nitrate inhibition, stress response, functional genomics, fitness profiling

## Abstract

Nitrate is an inhibitor of sulfate-reducing bacteria (SRB). In petroleum production sites, amendments of nitrate and nitrite are used to prevent SRB production of sulfide that causes souring of oil wells. A better understanding of nitrate stress responses in the model SRB, *Desulfovibrio vulgaris* Hildenborough and *Desulfovibrio alaskensis* G20, will strengthen predictions of environmental outcomes of nitrate application. Nitrate inhibition of SRB has historically been considered to result from the generation of small amounts of nitrite, to which SRB are quite sensitive. Here we explored the possibility that nitrate might inhibit SRB by a mechanism other than through nitrite inhibition. We found that nitrate-stressed *D. vulgaris* cultures grown in lactate-sulfate conditions eventually grew in the presence of high concentrations of nitrate, and their resistance continued through several subcultures. Nitrate consumption was not detected over the course of the experiment, suggesting adaptation to nitrate. With high-throughput genetic approaches employing TnLE-seq for *D. vulgaris* and a pooled mutant library of *D. alaskensis*, we determined the fitness of many transposon mutants of both organisms in nitrate stress conditions. We found that several mutants, including homologs present in both strains, had a greatly increased ability to grow in the presence of nitrate but not nitrite. The mutated genes conferring nitrate resistance included the gene encoding the putative Rex transcriptional regulator (DVU0916/Dde_2702), as well as a cluster of genes (DVU0251-DVU0245/Dde_0597-Dde_0605) that is poorly annotated. Follow-up studies with individual *D. vulgaris* transposon and deletion mutants confirmed high-throughput results. We conclude that, in *D. vulgaris* and *D. alaskensis*, nitrate resistance in wild-type cultures is likely conferred by spontaneous mutations. Furthermore, the mechanisms that confer nitrate resistance may be different from those that confer nitrite resistance.

## Introduction

Sulfate-reducing bacteria (SRB) are environmentally and industrially significant microorganisms that use sulfate as a terminal electron acceptor in anaerobic respiration. These anaerobes produce sulfide as the end product of sulfate respiration (Postgate, 1984). Sulfide is toxic to most organisms (Caffrey and Voordouw, 2010), and its production causes oil souring in the petroleum industry (Ligthelm et al., 1991; Sunde et al., 1993). Despite the undesirable features of this metabolic end product, SRB have been exploited in studies of heavy metal bioremediation (Jiang and Fan, 2008; Martins et al., 2009) because of the ability of sulfide to form insoluble complexes with heavy metals (Jalali and Baldwin, 2000). SRB also precipitate heavy metals by directly changing the metal redox state to a less soluble form (Lovley et al., 1993a,b; Lloyd et al., 1999; Chardin et al., 2003). The metabolism of SRB is studied, therefore, to understand how to minimize the detrimental economic effects of these bacteria and to maximize their positive metabolic traits.

Such traits have been studied extensively in *Desulfovibrio vulgaris* Hildenborough (DvH), a model SRB with a sequenced genome (Heidelberg et al., 2004). DvH has been examined under a variety of stress conditions, including elevated nitrite (He et al., 2006; Bender et al., 2007) or nitrate concentrations (Redding et al., 2006; He et al., 2010a), heat shock (Chhabra et al., 2006), high salt (Mukhopadhyay et al., 2006; He et al., 2010b), oxygen (Mukhopadhyay et al., 2007), or electron donor depletion (Clark et al., 2006). The data obtained help in the prediction of responses of SRB in heavy metal-contaminated sites, which also contain many chemicals that inhibit these bacteria. For example, nitrate concentrations can be greater than 100 mM at US nuclear weapon complexes overseen by the Department of Energy (Green et al., 2012), and these waste sites are also contaminated with heavy metals (Riley and Zachara, 1992). High nitrate inhibits the growth of DvH (He et al., 2010a). Although some SRB can also use nitrate as a terminal electron acceptor (McCready et al., 1983), nitrate is successfully used by the petroleum industry to control the growth of SRB and the oil souring that their sulfide production causes (Sunde and Torsvik, 2005). The mechanism of nitrate inhibition of SRB is still unclear. In the environment, at least part of the inhibition by nitrate is indirect: nitrate-reducing, sulfide-oxidizing bacteria produce nitrite that is toxic to SRB at much lower concentrations than is nitrate (Haveman et al., 2005; He et al., 2006). Furthermore, in oil wells, heterotrophic nitrate-reducing bacteria can compete with SRB for volatile fatty acid electron donors, further reducing the production of sulfide (Grigoryan et al., 2008). However, nitrate is also inhibitory to DvH in the absence of nitrate-reducing bacteria (Redding et al., 2006; He et al., 2010a).

It has been suggested that this pure culture nitrate inhibition is also a result of nitrite stress, since DvH itself may produce small amounts of nitrite from non-specific reduction of nitrate (Wall et al., 2007). In addition, high concentrations of nitrate could potentially induce a non-specific osmotic shock response in the bacteria (Wall et al., 2007). However, microarray data reveal few common gene expression changes among nitrate, nitrite, and sodium chloride stress conditions (He et al., 2010a). He et al. suggested that unique nitrate stress responses might account for these discrepancies (He et al., 2010a). Understanding the mechanism of nitrate inhibition of DvH and the genes involved in the nitrate stress response should facilitate the prediction and monitoring of the effectiveness of bioremediation strategies that employ SRB (Hazen and Stahl, 2006).

Past studies of the mechanisms of nitrate stress responses in DvH have relied primarily on transcript analyses (He et al., 2010a) and protein determination (Redding et al., 2006) techniques. However, mutant analysis is a more reliable method of determining gene essentiality in a particular stress condition (Price et al., 2013). Fitness profiling of many mutants en masse is a high-throughput approach complementary to classical genetic techniques that has allowed rapid annotation of genes (Deutschbauer et al., 2011). In this study, we used random transposon mutant fitness profiling in two completely sequenced (Heidelberg et al., 2004; Hauser et al., 2011) model SRB, *Desulfovibrio alaskensis* G20 (“G20,” formerly called *Desulfovibrio desulfuricans* G20) and DvH, to probe the molecular mechanisms of their nitrate stress responses. Because 58% of G20 genes (1954/3371) are also present in DvH, which has 3503 genes (http://www.microbesonline.org/), we predicted that the strains would have similar nitrate stress responses. Therefore, pools of DvH and G20 transposon mutants with mutations saturating the non-essential genes under permissive growth conditions were subjected to high concentrations of nitrate. Illumina sequencing or microarrays were used to locate the transposons in mutants surviving the nitrate exposure and, by comparison with mutants not exposed to stress, to identify genes essential for survival in high nitrate. Generally, those mutants lost from the stress treatment represent genes whose functions are needed for stress survival. From the fitness profiling reported here, surprisingly we identified mutants with dramatically increased fitness in nitrate stress conditions that we further analyzed in pure cultures. However, the same mutations did not confer resistance to nitrite. These results confirmed the predicted existence of unique nitrate-resistance mechanisms (He et al., 2010a) and suggested that environmental models of nitrate inhibition require expansion.

## Materials and methods

### Strains and media

The strains used in this study are listed in Table 1. All DvH and G20 strains were grown in defined MOLS4 medium [MO Basal Salts (Zane et al., 2010) with 60 mM sodium lactate and 30 mM sodium sulfate]. The medium used to grow DvH cultures was reduced with 1.2 mM sodium thioglycolate; whereas, the medium for G20 was reduced with 0.38 mM titanium citrate. DvH and G20 manipulations, including setup of growth kinetic studies, were done at about 25°C in an anaerobic growth chamber (Coy Laboratory Products, Inc., Grass Lake, MI) with an atmosphere of approximately 95% N_2_ and 5% H_2_. Optical densities (600 nm) were determined with a Genesys 20 spectrophotometer (Thermo Scientific, Waltham, MA).

**Table 1:** **Strains and plasmids used in this study**.

**Strain or plasmid**	**Genotype or relevant characteristics^a^**	**Sources and/or references**
***E. coli***		
α-Select (Silver Efficiency)	F^−^ *deo*R *end*A1 *rec*A1 *rel*A1 *gyr*A96 *hsd*R17(r^−^_k_, m^+^_k_) *sup*E44 *thi*-1 *pho*A Δ (*lac*ZYA-*arg*F)U169 Φ 80*lac*ZΔ M15 λ^−^	Bioline
***D. alaskensis***		
G20	Spontaneously nalidixic acid-resistant derivative of *Desulfovibrio desulfuricans* G100A lacking the endogenous cryptic 2.3-kb plasmid, pBG1	Wall et al., 1993
***D. vulgaris***		
ATCC29579	Wild-type *D. vulgaris* Hildenborough (pDV1); 5-FU^s^ (Parent for GZ strains)	ATCC
JW710	WT Δ *upp* (pDV1); 5-FU^r^ (used as “WT” control for DvH growth kinetics in this study; parent strain for deletions)	Keller et al., 2009)
JW3311	JW710 Δ DVU0916::(*npt upp*); Km^r^; 5-FU^s^ (Δ*rex* marker exchange)	This study
GZ9685	DVU0245-773::Tn*5*-RL27;insertion at bp 773/1110 for the gene; Km^r^	Wall laboratory
GZ12997	DVU0246-111::Tn*5*-RL27;insertion at bp 111/2235 for the gene; Km^r^	Wall laboratory
GZ2640	DVU0247-211::Tn*5*-RL27;insertion at bp 211/360 for the gene; Km^r^	Wall laboratory
GZ12015	DVU0250-427::Tn*5*-RL27;insertion at bp 427/588 for the gene; Km^r^	Wall laboratory
GZ10694	DVU0251-80::Tn*5*-RL27;insertion at bp 80/963 for the gene; Km^r^	Wall laboratory
GZ2179	Genome position 658487::Tn*5*-RL27; insertion at intergenic region 327 bp upstream of VIMSS209534, DVU0590; Km^r^ (Control strain for transposon mutant growth kinetics)	Wall laboratory
**PLASMIDS**		
pMO719	pCR8/GW/TOPO containing SRB replicon (pBG1); Sp^r^; source of Sp^r^ and pUC *ori* fragment for marker exchange suicide plasmid construction	Keller et al., 2009
pMO746	*upp* in artificial operon with *npt* and Ap^r^-pUC *ori*; P_*npt*_-*npt*-*upp*; Km^r^; 5-FU^s^; for marker exchange suicide plasmid construction	Parks et al., 2013
pMO9075	pMO719 containing P_*npt*_ for constitutive expression of complementation constructs; pBG1 stable SRB replicon; Sp^r^	Keller et al., 2011, 2014
pMO3311	Sp^r^ and pUC *ori* from pMO719 plus 1630 bp upstream and 1590 bp downstream DNA regions from DVU0916 (*rex)* flanking the artificial operon of P_*npt*_-*npt-upp* from pMO746; for marker exchange deletion mutagenesis; Sp^r^ and Km^r^	This study
pMO3313	pMO9075 with DVU0916 (*rex*) constitutively expressed from P_*npt*_	This study
pRL27	Tn*5*-RL27 (Km^r^-*ori*R6 K) delivery vector; for transposon mutagenesis of DvH strains	Larsen et al., 2002

a Km, kanamycin; Sp, spectinomycin; Ap, ampicillin; 5-FU, 5-fluorouracil; superscript “r” or “s,” resistance or sensitivity.

### Growth kinetics

Growth kinetic studies with WT G20 were carried out essentially as described below for the G20 fitness profiling, with the following exceptions: No kanamycin was used with the WT cells, and each wild-type G20 freezer stock was pelleted to reduce carryover of glycerol used as the cryoprotectant before inoculation of starter cultures. For all DvH growth kinetics, 5 mL MOLS4 cultures (with 1.2 mM sodium thioglycolate) were started by inoculation with the pelleted cells from a freezer stock. Geneticin (G418) sulfate (400 μg/mL) or spectinomycin dihydrochloride pentahydrate (100 μg/mL) were added to DvH cultures where indicated. Each condition tested was prepared as 14.5 mL of inoculated culture plus 1 mL deionized water (for “no additions” controls) or inhibitory salts (sodium nitrate, sodium nitrite) dissolved in deionized water. Aliquots of 5.1 mL from this 15.5 mL culture were grown as triplicates in 27-mL anaerobic tubes, each capped with a butyl rubber stopper and grown at 34°C. All G20 and DvH inocula were grown to late exponential or stationary phase. 100 mM sodium nitrate was used for experiments with DvH, compared with 150 mM for G20 experiments, due to the greater sensitivity of DvH to nitrate. With a few exceptions, all growth kinetics experiments were repeated at least twice with triplicates in each experiment. Triplicate growth experiments for the DVU0250 transposon mutant and intergenic control transposon mutant experiments were done once. Triplicates of the Δ*rex* mutant in the presence of nitrite were also grown once.

### G20 transposon mutant fitness studies

Fitness data were collected with two pools of G20 transposon insertion mutants (4069 and 4056 mutants, respectively) that will be described in more detail elsewhere (Kuehl et al., in revision). Briefly, 1174 strains are present in both pools, leaving 6951 strains that are present only once in the complete library. 498 genes are represented only once in the library. 571 genes are represented twice in the library; that is, either a single strain is present in each of the pools or two different strains with a transposon insertion in the same gene are present in the library. 1341 genes are represented in the library three or more times. A total of 2410 unique genes and 212 unique intergenic regions are represented. Thus, about 71% of G20 genes are represented in the library, providing excellent coverage of non-essential genes. Transposon insertions were mapped to the genome by a two-step arbitrary PCR as described previously (Oh et al., 2010). Each mutant has a “TagModule” that contains two different variable segments, an “uptag” and a “downtag”(Oh et al., 2010). Within each pool, each strain has a unique TagModule, so that the abundance of the TagModule is a proxy for the abundance of that strain. Only the uptags are amplified from the “uptag pool,” Pool 1 (4069 strains) and only the downtags are amplified from the “downtag pool” Pool 2 (4056 strains). Amplified tags from both pools can be hybridized to the same array because only one tag (up or down) from a TagModule has been shown to be necessary for accurate quantification of strain abundance and there is no overlap of tags in the two pools (Oh et al., 2010; Deutschbauer et al., 2011). Each pool was grown overnight to late log phase (OD about 0.87) in about 10 mL MOLS4 medium amended with titanium citrate (0.38 mM) as reductant and kanamycin (800 μg/mL). 750 μL of each pool was added to 15 mL MOLS4 + 1650 μL sterile MO Basal salts (Zane et al., 2010) or salt (sodium nitrate, sodium chloride, etc.) dissolved in MO Basal Salts. Each amended medium plus mutants (17.4 mL) was aliquoted into three 27-mL anaerobic tubes, about 5.8 mL per tube, each capped with a butyl rubber stopper and grown at 34°C. When the cultures had reached stationary phase (OD > 1), 0.5 mL from each control or stress condition was pelleted and processed as described previously; that is, genomic DNA was extracted (Deutschbauer et al., 2011), and the uptags and downtags were PCR amplified, hybridized to an Affymetrix 16K TAG4 array, and scanned (Pierce et al., 2007). The number of doublings of the population was estimated by using the doubling in OD_600_ to approximate doubling of the cell population.

Fitness data for G20 were analyzed as described for similar experiments with *Shewanella oneidensis* MR-1 (Deutschbauer et al., 2011), with slight modifications (Price et al., 2013). Briefly, strain fitness = log_2_(END/START), where those values (“END” and “START”) are averages of the gene location-specific uptag and downtag log_2_ intensities. Mutant strains with low START values were excluded, leaving measurements for 3726 strains in Pool 1 and 3865 strains in Pool 2. Strain fitness was normalized across the genome so that the median was 0; this was done separately for the two pools. Since a gene could be mutated at different sites, gene fitness was calculated as the average fitness of strains with mutations in a particular gene. Gene fitness was normalized to remove the effect of chromosomal position on gene fitness and to set the mode of fitness values to zero, as previously described (Price et al., 2013). One difference from the previously described protocol (Price et al., 2013) was that only one Affymetrix chip was used per experiment; up- and downtags were hybridized to the same array. The results of the “MOLS4 no stress” condition were similar to the LS4D controls described previously (Price et al., 2013), including similarly “sick” auxotrophs that would be expected in a defined medium when compared with lactate-sulfate medium containing yeast extract. Two quality metrics were used for each experiment. Strain correlation (Table S1) is the correlation of the strain fitness values for the same strains between the two pools. Operon correlation (Table S1) is the correlation of gene fitness values between adjacent genes predicted (http://www.microbesonline.org/) to be in the same operon. The low quality metrics for the sodium nitrate and potassium nitrate conditions (Table S1) reflect the predominance of a few strains in the culture; essentially only the data for these few strains is biologically meaningful, while the majority of strains did not have the opportunity to grow at all before the culture reached stationary phase. The complete data from these experiments are available at http://www.microbesonline.org/.

### DvH TnLE-seq fitness studies

This nitrate resistance study was one of five multiplexed TnLE-seq pools that were part of a protocol described elsewhere (Fels et al., 2013). One advantage of this method is that individual mutants do not need to be isolated and confirmed, as in a catalogued library. They also do not need to be frozen en masse and recovered from the freezer. Rather, hundreds of thousands of unique transposon mutations are created by conjugation at the beginning of each experiment. The only experimental difference between this study and those published was the addition of 100 mM nitrate to the MOLS4 defined medium for growth of the mutant pool. As expected given the strong stress of 100 mM nitrate, this pool was delayed by about 92 h in reaching an OD_600_ of 0.4 compared with only about 40 h in the defined MOLS4 medium without nitrate. However, the nitrate pool was harvested and the fitness values were determined as previously described (Fels et al., 2013). The total number of cells in the final 500 mL culture (1 × 10^11^ cells) was determined by plating for individual colony-forming units, as previously described (Fels et al., 2013). The number of doublings of the culture was estimated by assuming that only the genes with log_2_ fitness scores >0 (38 genes) contributed significantly to the final population. Therefore, the number of unique insertions in these genes (1904) was considered to be number of cells in the starting pool. The complete data from these experiments are available at http://desulfovibriomaps.biochem.missouri.edu/fitness/.

### Plasmid and strain construction

Genomic DNA from DvH was isolated with the Wizard® Genomic DNA Purification Kit (Promega, USA). Plasmids were isolated from both *E. coli* and DvH with the GeneJET Plasmid Miniprep Kit (Fermentas, Thermo Scientific, Glen Burnie, MD). All primers were obtained from Integrated DNA Technologies (Coralville, IA). The pMO3311 and pMO3313 plasmids were constructed by Sequence- and Ligation-Independent Cloning (SLIC) (Li and Elledge, 2007). PCR products from template plasmids were agarose gel-purified to reduce transformation of the parent plasmid. All products were cleaned with a Wizard® SV Gel and PCR Clean-up System (Promega, USA) before the SLIC procedure. The plasmids were constructed by amplification of DNA regions (Table S2) with Herculase II polymerase (Agilent cat# 600675), as previously described for a similar procedure (Parks et al., 2013). DNA products were transformed into Silver Efficiency α-Select *E. coli* cells (Bioline) and plated on solidified LC medium (Zane et al., 2010). Electroporation procedures were similar to those previously described (Keller et al., 2011) with electroporation parameters 1500 V, 250 Ω, and 25 μF. Cells recovered overnight after electroporation were plated on MOYLS4 with 1.2 mM thioglycolate as reductant and about 0.2% (wt/vol) yeast extract. Sequence confirmation of the mutagenic cassette and the complementing gene was performed at the University of Missouri DNA Core Facility (http://www.biotech.missouri.edu/dnacore/).

### Nitrate determination

A scaled-down version of a previously described colorimetric method (Cataldo et al., 1975) was used to determine nitrate concentrations. Briefly, 200 μL of salicylic acid solution (1 g salicylic acid dissolved in 20 mL of approximately 98% [vol/vol] sulfuric acid) was added to each 25 μL sample that had been diluted 25-fold in deionized water. This was mixed and incubated 20 min at room temperature and then 4.75 mL of 2 M NaOH was added to each tube. Absorbance at 410 nm was measured with a Genesys 20 spectrophotometer (Thermo Scientific, Waltham, MA). *R*^2^ for a standard curve was >0.99, instrument detection limit 0.1 ± 0.1 mM.

### Protein determinations

Whole cell protein concentrations were determined with the Bradford assay (Bradford, 1976) with bovine serum albumin as the standard. Absorbance at 595 nm was measured with a Genesys 20 spectrophotometer (Thermo Scientific, Waltham, MA).

## Results

### Response of DvH to nitrate exposure

**Figure 1:**
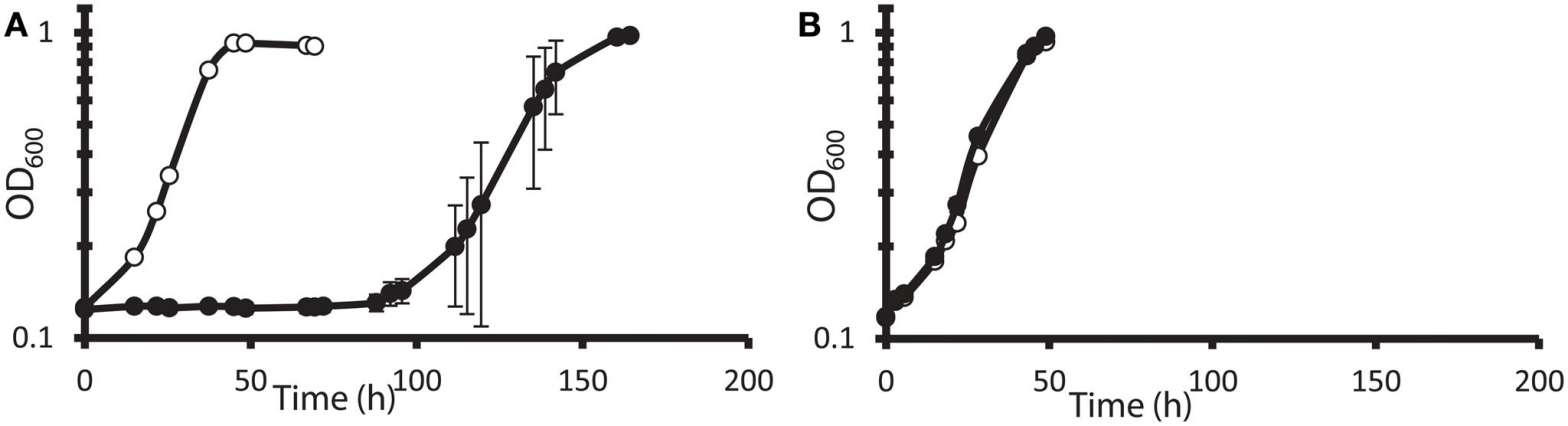
**Growth and subculture of wild-type *D. vulgaris* Hildenborough (JW710) in lactate-sulfate medium with inhibitory nitrogen species. (A)** Growth of DvH with no additions (°) or 100 mM sodium nitrate (•) **(B)** Growth of DvH subcultured from original 100 mM nitrate culture shown in **(A)** no additions (°), 100 mM sodium nitrate (•). Approximately 6–6.5% (vol/vol) inocula were used for the original culture and subcultures. Readings reflect averages of three samples, and errors bars show standard deviations.

It has been reported (Elias et al., 2009; He et al., 2010a) that DvH cells can grow rapidly and abundantly after a long lag phase in high nitrate concentrations. It was unclear, however, whether this rapid growth was due to elimination of the toxic nitrate, some modification of cell metabolism allowing adaptation to the continued presence of nitrate, or outgrowth of preexisting nitrate-resistant mutants. Furthermore, it was not known whether the cells that grew in nitrate had a growth advantage over naïve cells when subcultured into a fresh medium amended with nitrate. To test this, JW710 (Table 1), the parent for making marker exchange and markerless deletion strains (Keller et al., 2009), was used. JW710 will therefore be referred to as the wild-type control for all DvH growth kinetics in this study. This wild-type control was grown in lactate-sulfate medium amended with 100 mM nitrate (Figure 1A).

It was determined that, at the end of growth in the presence of 100 mM nitrate, no gross consumption of nitrate was detected (Table 2). The persistence of the nitrate suggested that the ability of DvH to grow in the presence of 100 mM nitrate was due to adaptation to nitrate or outgrowth of spontaneous mutants, rather than a detoxification of nitrate itself. To further confirm this lack of nitrate metabolism, nitrite was measured (American Public Health Association, 1992) during the lag/inhibition phase for JW710 cells exposed to 100 mM nitrate and the nitrite concentration was below 15 ± 5 μM (data not shown). This nitrite concentration is below the concentration (40 μM) reported to inhibit plated single colonies of *D. vulgaris* (Haveman et al., 2004). Thus, secondary production of nitrite is not likely the cause of nitrate sensitivity. To begin to test whether the nitrate adaptation was due to a reversible gene regulation or to successful growth of spontaneous mutants in the culture, nitrate-adapted strains were subcultured back into medium with or without nitrate. We found that nitrate-stressed cultures grew without a prolonged lag and maintained nitrate resistance when subcultured into fresh medium with 100 mM nitrate (Figure 1B). This resistance continued over the course of three subcultures (data not shown). Further, even nitrate-resistant cultures that were subcultured into medium lacking nitrate retained nitrate resistance when subcultured back into 100 mM nitrate (data not shown). As with the original exposure to nitrate, no gross consumption of nitrate was observed over the course of the subcultures (Table 2). We suggest that spontaneous mutations in the culture lead to increased nitrate resistance of some cells which then predominate in the population.

**Table 2:** **Nitrate concentrations from stationary phase cultures of *D. vulgaris* Hildenborough grown in MOLS4 medium amended as indicated**.

**Subculture^a^**	**Inoculum history**	**Amendment**	**[NO^−^_**3**_]^b^ in mM**
0-A^c^	Lactate/SO^2−^_4_	none	not measured
0-B^c^	Lactate/SO^2−^_4_	100 mM NO^−^_3_	101 ± 3
1-A^d^	From 0-B	none	8 ± 1
1-B^d^	From 0-B	100 mM NO^−^_3_	102 ± 3
2-A	From 1-B	none	6 ± 2
2-B	From 1-B	100 mM NO^−^_3_	99 ± 1
3-A	From 2-A	none	not detected
3-B	From 2-A	100 mM NO^−^_3_	101 ± 7
3-C	From 2-B	none	8 ± 2
3-D	From 2-B	100 mM NO^−^_3_	103 ± 5

a Inocula were 6.5% (vol/vol).

b Concentrations determined from triplicate determinations with standard deviations shown.

c Growth curves in Figure 1A.

d Growth curves in Figure 1B.

### Fitness profiling with G20

To test what mutations might be causing this nitrate resistance, we employed transposon mutant fitness profiling. A catalogued transposon mutant library, which enables high-throughput phenotypic screening, had been generated in G20 prior to that produced in DvH (Price et al., 2013). Each mutant strain in the library is identified by two unique DNA barcode sequences or “tags,” the “up” tag and the “down” tag (Oh et al., 2010; Deutschbauer et al., 2011). Strain abundance is measured by reading the abundance of the barcodes through fluorescence in microarrays made to detect the barcodes (Pierce et al., 2006, 2007).

We predicted that comparison of fitness profiles of nitrite- and nitrate-stressed G20 cells would reveal mutants with responses unique to nitrate. That is, fitness profiling in the two conditions would allow us to see which mutants differentially increased in relative abundance during a pooled growth competition and which decreased. The fitness of a particular strain is calculated as log_2_ of the ratio of the relative abundance of the strain after growth competition to the relative abundance of the strain before growth competition. Therefore, if the relative abundance of a particular strain in the pool remained the same before and after stress, its fitness would be equal to zero (Oh et al., 2010):

$$\begin{array}{l} \text{Fitness of a G20 mutant}\\ \;=\log_{2}\left(\frac{\text{barcode microarray signal from cells after stress}}{\text{barcode microarray signal from cells prior to stress}}\right) \end{array}$$

If a strain decreased in relative abundance after the stress condition because it was outcompeted or unable to cope with the stress, it would have negative (<0) fitness. If its relative abundance increased, it would have positive (>0) fitness. For the pools, the fitness calculated for a particular gene, referred to as the “mean log ratio,” is expressed as log_2_ of the average fitness of strains with a mutation in that particular gene.

**Figure 2:**
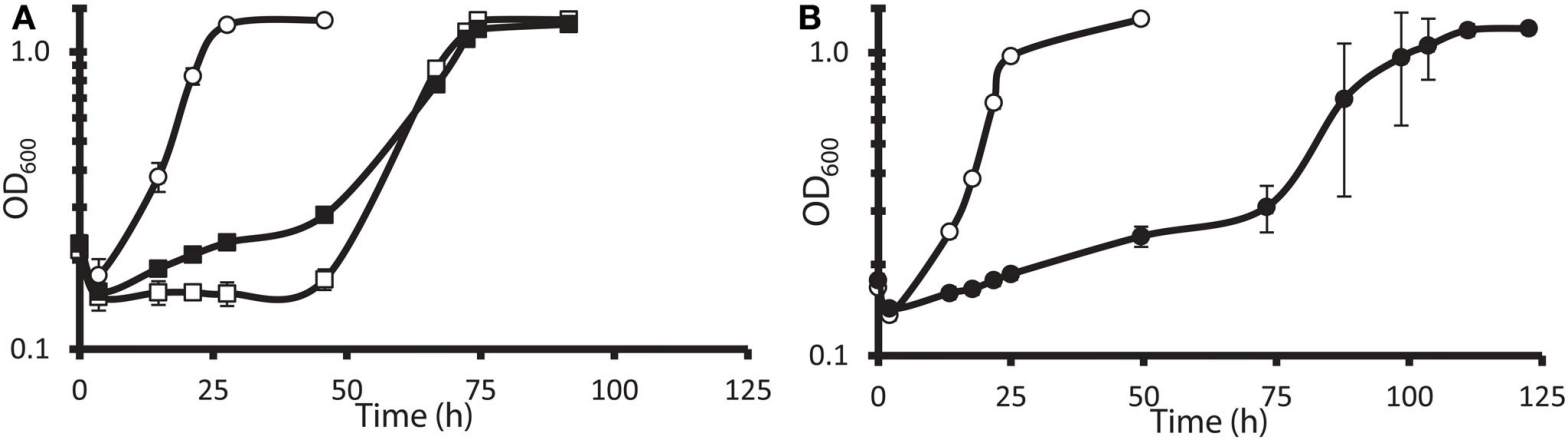
**Growth of wild-type *D. alaskensis* G20 in lactate-sulfate medium with inhibitory nitrogen species. (A)** Growth of G20 with no additions (°), 150 mM sodium nitrate (□), or 150 potassium nitrate (◾). **(B)** Growth of G20 with no additions (°) or 0.25 mM sodium nitrite (•). Approximately 4.5% (vol/vol) inocula were used. Readings reflect averages of three samples, and errors bars show standard deviations.

**Figure 3:**
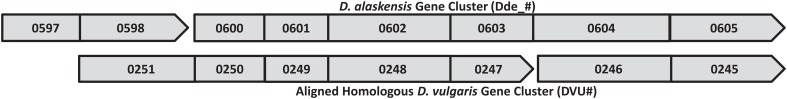
***Desulfovibrio* nitrate resistance gene cluster**. Operon predictions were from http://microbesonline.org/; boxes represent predicted genes, arrows indicate direction of transcription, and contiguous boxes ending in an arrow represent predicted operons.

The G20 pools were grown in lactate-sulfate medium amended with 150 mM NaNO_3_, 150 mM KNO_3_, 150 mM NaCl, 150 mM KCl, or 0.25 mM NaNO_2_. NaCl and KCl conditions were osmotic controls; KNO_3_ vs. KCl allowed a control for anion specificity. Concentrations of 150 mM nitrate and 0.25 mM nitrite were chosen because these concentrations severely but not completely inhibited wild-type G20 (Figures 2A,B). Since a long lag phase had been observed before exponential growth of nitrate-stressed cultures of both DvH and G20 in lactate-sulfate conditions (Figures 1A, 2A), it was reasonable to hypothesize that spontaneous mutants in wild-type cultures were selected in the population after the lag. Therefore, this hypothesis was confirmed when we found that, in the transposon mutant pools, several mutant strains predominated in cultures growing in the presence of nitrate (Table 3; Table S3). That is, transposon insertion conferred a growth advantage, and therefore a high fitness, on these particular strains in the nitrate stress condition. The top 10 genes interrupted in the strains that grew abundantly in sodium nitrate had fitness values (mean log ratios) greater than 2 in that condition, but fitness values less than 0.25 in sodium chloride, potassium chloride, sodium nitrite, and in the absence of stress (Table S3). In contrast, interruption of those same genes was advantageous in both sodium nitrate and potassium nitrate (Table S3). Growth of the mutants in both salts of nitrate supports the specificity of the nitrate anion as the driver for selection of these resistant mutants. The interrupted gene conferring the highest fitness in sodium nitrate was Dde_2702 (Table 3), a gene annotated as encoding Rex, a redox-sensing regulatory protein (Ravcheev et al., 2012). Particularly surprising was the high fitness conferred by mutation of a cluster of poorly annotated genes, Dde_0597 through Dde_0605, hereafter called the “nitrate cluster.” Both the *rex* gene and the nitrate cluster (Table 3) have homologs in DvH. Because of these homologies, including shared synteny of the nitrate cluster in G20 (Figure 3), it seemed reasonable that mutations of the homologs in DvH would confer similar nitrate-resistant phenotypes.

**Table 3:** ***Desulfovibrio* genes interrupted in strains with high fitness in lactate-sulfate conditions amended with sodium nitrate**.

**G20 gene (Dde)**	**fitness^a^**	**DvH homolog (DVU)**	**fitness^b^**	**Annotations**
2702	4.23	0916	3.81	AT-rich DNA binding protein (COG2344); Transcriptional repressor, redox-sensing, Rex (IPR022876)
0597	2.25	no homolog	No data	Uncharacterized protein conserved in archaea (COG2043); Protein of unknown function DUF169 (IPR003748)
0598	3.01	0251	11.44	Transmembrane protein TauE like (IPR002781); predicted permease (COG0730); sulfite exporter TauE/SafE (pfam01925)
0600	2.88	0250	−5.82	Conserved hypothetical protein
0601	3.51	0249	3.86	PtxB, putative (http://microbesonline.org/); ABC-type phosphate/phosphonate transport system, periplasmic component (COG3221); outer membrane-associated homodimer (Walian et al., 2012)
0602	2.34	0248 (pseudo-gene)	1.43	Signal transduction histidine kinase (COG5002); PAS fold (IPR013767); ATPase-like, ATP-binding domain (IPR003594); HAMP linker domain (IPR003660); PAC motif (IPR001610)
0603	3.08	0247	9.14	Signal transduction response regulator, receiver domain (IPR001789); CheY-like superfamily (IPR011006); ntrX (http://microbesonline.org/)
0604	3.41	0246	2.18	Pyruvate phosphate dikinase, PEP/pyruvate-binding (IPR002192); PEP-utilizing enzyme, mobile domain (IPR008279); ATP-grasp fold, subdomain 1 (IPR013815); ATP-grasp fold, subdomain 2 (IPR013816)
0605	2.08	0245	−6.04	Protein-tyrosine/Dual-specificity phosphatase (IPR000387)

a *$\left(\frac{barcode\;microarray\;signal\;from\;cells\;after\;stress}{barcode\;microarray\;signal\;from\;cells\;prior\;to\;stress}\right)$; fitness of stationary-phase G20 cultures grown for about 3.3 doublings (about 63 h) in lactate-sulfate medium amended with 150 mM sodium nitrate*.

b *$\left[\left(\frac{\#\;insertions\;in\;gene}{length\;of\;gene}\right)/\left(\frac{\#\;insertions\;in\;all\;genes}{length\;of\;all\;genes}\right)\right]$; fitness determined from mid-log phase DvH cultures grown for about 25.5 doublings (92 h) in lactate-sulfate medium amended with 100 mM nitrate*.

### Fitness profiling with DvH

In order to test the hypothesis that both G20 and DvH used the same mechanisms for nitrate resistance, we had the opportunity to employ a different high-throughput fitness profiling method, Transposon Liquid Enrichment sequencing (TnLE-seq). This method (Fels et al., 2013) is based on deep sequencing of random transposon mutations to query DvH. TnLE-seq is a modification of the HITS (High-throughput Insertion Tracking by deep Sequencing) (Gawronski et al., 2009), Tn-seq (Van Opijnen et al., 2009), and TraDIS (Transposon Directed Insertion-site Sequencing) (Langridge et al., 2009) methods. However, the TnLE-seq method developed for DvH is especially well-adapted to oxygen-sensitive bacteria that have low electroporation efficiency (Fels et al., 2013). The mutated culture is grown in control vs. stress conditions, and deep sequencing then determines the abundance and location of mutations at the end of growth. Because of the differences in methods, the calculation of fitness is also different from that of the mutant library experiment (Fels et al., 2013). The fitness value shown below is in log_2_R format, for easier comparison with the G20 pools:

$$\begin{array}{l} \text{Fitness of a DvH gene=}\log_{\text{2}}[\left(\frac{\#\text{ insertions in gene}}{\text{length of gene}}\right)\text{/}\\ \;\left(\frac{\#\text{ insertions in }all\text{ genes}}{\text{length of }all\text{ genes}}\right)] \end{array}$$

As previously described (Fels et al., 2013), fitness was calculated from insertions only in the 5–85% region of the coding sequence of genes, as such insertions are more likely to impair the function of gene products. As with the G20 pool described above, negative fitness indicates a fitness defect, whereas positive fitness indicates that the mutation confers a fitness advantage in that particular condition. For nitrate stress, the transposon mutants were grown in lactate-sulfate medium amended with 100 mM sodium nitrate. As expected, the results showed that mutations in a predicted *rex* gene annotated as encoding a transcriptional regulator (DVU0916) as well as mutations in homologs of the G20 “nitrate cluster” (DVU0251, DVU0249, DVU0247, and DVU0246) conferred fitness values among the ten highest values (Table 3; Table S4). In fact, mutation of DVU0251 led to the highest fitness value, 11.44, or 2780-fold. Essentially, there was a “jackpot effect” in which a small percentage of mutants predominated in the population, a consistent result between the DvH and G20 fitness experiments. Despite these consistencies, in-depth, individual mutant analysis was necessary to confirm and elucidate the results of high-throughput fitness profiling (Deutschbauer et al., 2011).

### Confirmation of fitness profiling with individual mutants

**Figure 4:**
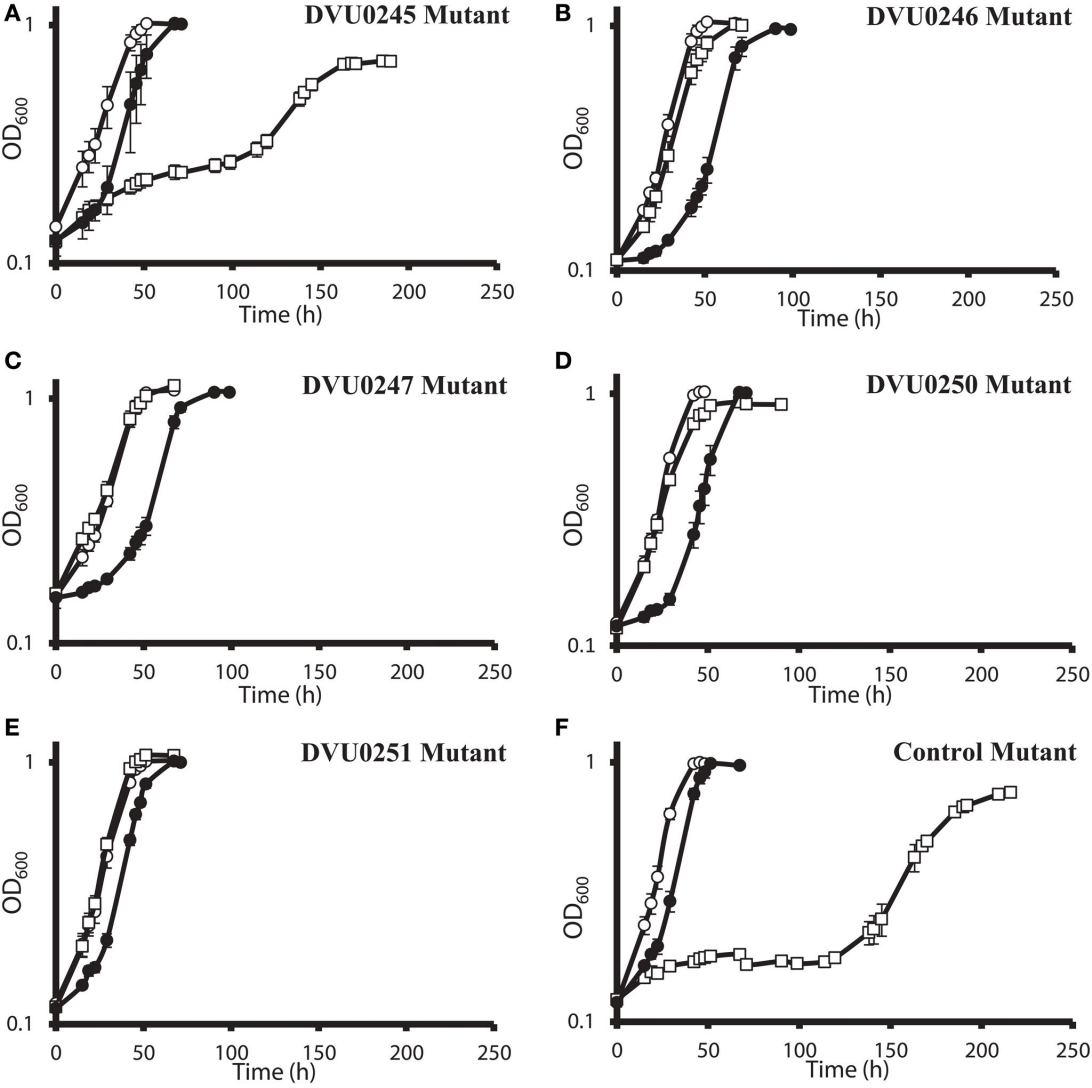
**Growth of *D. vulgaris* Hildenborough transposon mutants in lactate-sulfate medium with inhibitory nitrogen species**. Growth of the DVU0245 **(A)**, DVU0246 **(B)**, DVU0247 **(C)**, DVU0250 **(D)**, DVU0251 **(E)**, and intergenic transposon control **(F)** mutants in the presence of no additions (°), 100 mM sodium nitrate (□), or 1 mM sodium nitrite (•). Approximately 5.3% (vol/vol) inocula were used. Optical density readings reflect averages of three samples, and errors bars show standard deviations.

DvH was chosen for confirmation studies because a catalogued transposon mutant library of DvH was also available, in-frame deletion mutants can be made with greater facility (Keller et al., 2009), and complementation of mutants is readily accomplished. For an initial confirmation of the physiological relevance of the “nitrate cluster” to nitrate resistance, we determined growth kinetics of five DvH isolated mutants with transposon insertions in genes in that cluster (Figures 4A–E). The control strain used had a transposon at an intergenic position 327 base pairs upstream of a gene encoding a “random” hypothetical protein, DVU0590, and, therefore, was not predicted to be involved in nitrate stress responses. We found that the mutants with transposon insertions in DVU0246, DVU0247, DVU0250, and DVU0251 grew with indistinguishable kinetics with or without 100 mM nitrate. In contrast, 100 mM nitrate inhibited the control and the DVU0245 transposon mutants (Figures 4A–F). Inhibition of the DVU0245 mutant in high nitrate is consistent with the low fitness of the DVU0245 mutant in nitrate (fitness −6.04, Table 3) and consistent with the inhibition of a deletion mutant of DVU0245 (data not shown). None of the mutants grew better than the control strain in 1 mM sodium nitrite (Figures 4A–F). We interpret these results to mean that the growth advantage of these mutants is specific to nitrate and not simply an advantage in the presence of inhibitory nitrogen species.

The results of the G20 and DvH fitness studies indicated similar responses of homologous genes. In the G20 results, the predicted *rex* mutant had the highest fitness (Dde_2702). The fitness score for the DvH *rex* mutant was also in the top ten fitness scores, along with four DvH “nitrate cluster” (DVU0251, DVU0247, DVU0249, DVU0246) mutants (Table 3; Table S2). However, both the DVU0245 and the DVU0250 mutants had low fitness in nitrate, whereas mutants of their G20 homologs had high fitness in nitrate (Table 3). The nitrate resistance of the pooled vs. the isolated DvH mutants was also not entirely consistent. The rapid growth of the isolated DVU0250 mutant (Figure 4D) in the presence of nitrate was unexpected because of the low TnLE-seq fitness conferred in the same condition by mutation of DVU0250 (Table 3). In addition, preliminary growth kinetic data (not shown) suggest that the transposon mutant of DVU0248, which is annotated as a pseudogene in DvH, has little or no growth advantage over the control strain in the presence of 100 mM nitrate. In contrast, in the pooled experiment the DVU0248 mutations conferred positive fitness in nitrate (Table 3). While more data will be needed either to confirm or to change the annotation of DVU0248 as a pseudogene, we suggest that similar nitrate-resistance mechanisms are operating in G20 and DvH. The discrepancies between pooled and individual mutant studies confirm the need for follow-up studies of high-throughput experiments.

**Figure 5:**
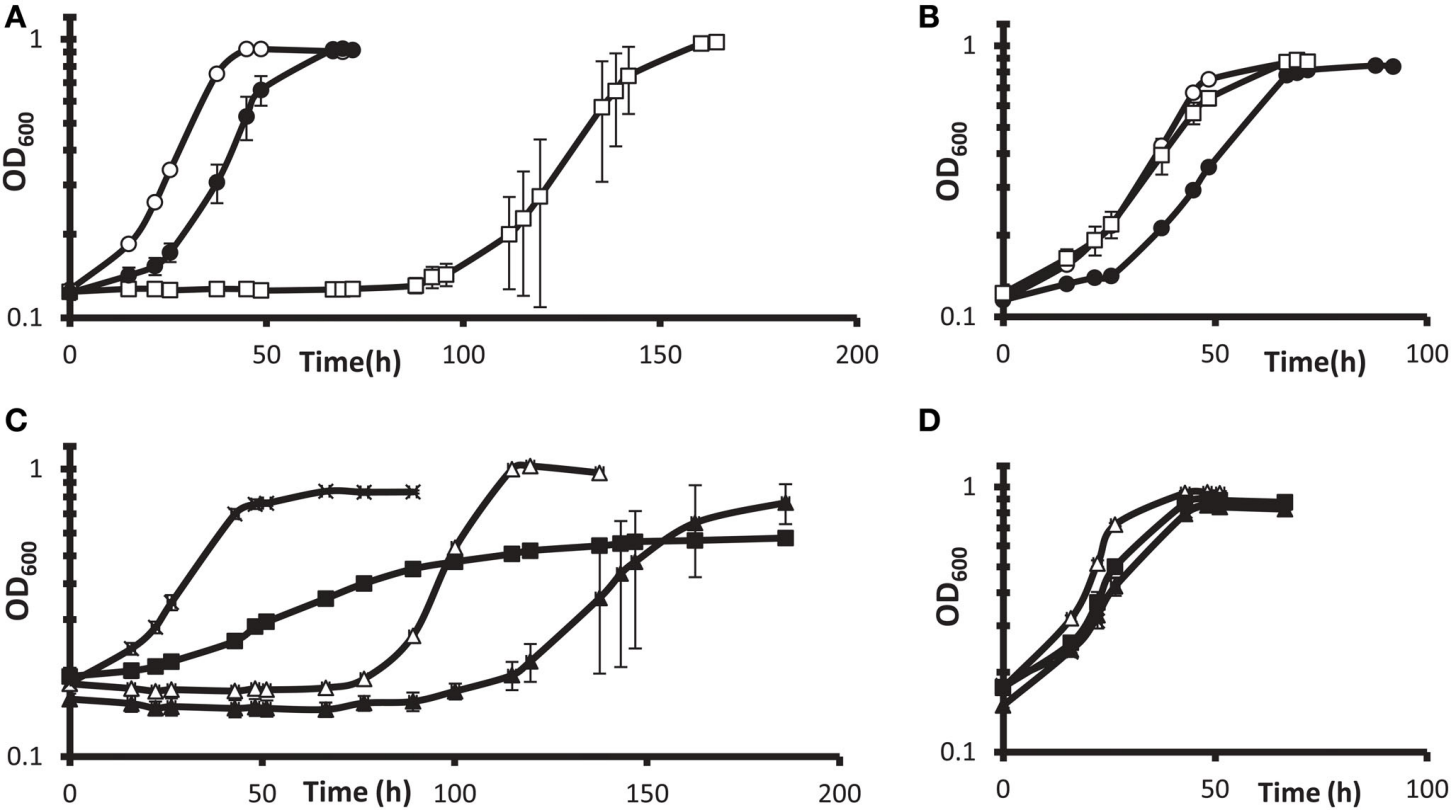
**Growth of DvH wild-type vs. Δ *rex* mutant in lactate-sulfate medium with inhibitory nitrogen species. (A,B)** Growth of the “wild-type” parental strain JW710 **(A)** vs. Δ *rex* **(B)** mutant in the presence of no additions (°), 100 mM sodium nitrate (□), or 1 mM sodium nitrite (•). Approximately 6% (vol/vol) inocula were used. **(C,D)** show four strains grown with 100 mM nitrate **(C)** or no additions **(D)**. Wild-type with empty vector [JW710(pMO9075)],(Δ); wild-type with *rex* overexpression plasmid [JW710(pMO3313)], (▴); Δ *rex* strain with empty vector [JW3311(pMO9075)], (x); Δ *rex* strain with *rex* complement plasmid [JW3311(pMO3313)], (◾). Approximately 7.5% (vol/vol) inocula were used. Readings reflect averages of three samples, and errors bars, which were often within the symbols, show standard deviations.

Such follow-up was pursued with gene deletion and complementation of the DvH gene encoding the predicted transcription regulator Rex. Interruption of the *rex* gene conferred the highest fitness in G20 but not in DvH (Table 3). We found that a deletion of DvH *rex* (DVU0916) had a clear advantage over the JW710 parent strain in lactate-sulfate medium with 100 mM added nitrate (Figures 5A,B). Like the “nitrate cluster” transposon mutants described above (Figures 4A–F), the *rex* mutant is not demonstrably resistant to nitrite (Figures 5A,B). Interestingly, when the mutant was complemented with *rex* expressed from a constitutive promoter, the phenotype in the presence of 100 mM nitrate was different from either parent or mutant phenotypes (Figure 5C). In contrast, the parent strain with *rex* overexpressed appeared to be at least as sensitive to nitrate as the parent strain (Figure 5C). The unique phenotype of the complemented mutant may result from some of the bacterial population losing the plasmid containing the complemented *rex* gene, in spite of antibiotic selection. While spectinomycin selects for plasmid retention, nitrate should select for plasmid loss in a Δ*rex* strain grown in high nitrate. Cells containing the plasmid may produce enough of the antibiotic-modifying enzyme to confer sufficient resistance to allow other cells to survive without containing the plasmid. If this is the case, then the Δ*rex* cells containing the plasmid should grow slowly while those which have lost the plasmid should grow more rapidly in the presence of 100 mM nitrate. The result would be a population growth rate in-between that seen for wild-type vs. Δ*rex* strains. Indeed, the phenotype of the complemented Δ*rex* strain exhibits this growth property (Figure 5C). Finally, nitrate concentrations in the cultures with empty vector or *rex* complementing plasmids (Figures 5C,D) were measured colorimetrically at the end of growth. As with the wild-type cultures described above, gross consumption of nitrate was not detected for any of these strains (data not shown). This is evidence of genuine nitrate resistance in these strains. Taken together, these growth and gene fitness data support transcriptomic predictions that nitrate stress responses involve mechanisms independent of nitrite stress responses (He et al., 2010a).

## Discussion

Whereas Elias et al. (2009) and Bender et al. (2007) reported mutations that led to increased sensitivity to both nitrate and nitrite, here we report the unexpected discovery of DvH mutants with increased resistance to nitrate but not nitrite. The data presented confirm that the *rex* deletion and the “nitrate cluster” transposon mutants, the top candidates from fitness profiling, confer resistance to nitrate in DvH. Such resistance also developed in the non-mutagenized DvH parental strain after subculture from 100 mM nitrate (Figure 1B), likely as a result of the outgrowth of preexisting spontaneous nitrate-resistant mutants. The lack of nitrate metabolism of DvH is consistent with a report that 10 mM nitrate did not noticeably inhibit DvH (Haveman et al., 2004). Furthermore, DvH has been shown to reduce nitrite (Haveman et al., 2004), but not nitrate.

The ability of mutations in a subset of non-essential genes to confer nitrate resistance may in part account for the recently reported fluctuating sulfide levels produced by SRB in a bioreactor inoculated with oil from a Canadian oil field (Callbeck et al., 2013). In this bioreactor, sulfide production was completely inhibited during pulses of 100 mM nitrate. However, after each pulse, sulfide production resumed, indicating that some SRB persisted in the presence of the nitrate (Callbeck et al., 2013). The results of the work presented here suggest that persistence of SRB in nitrate-treated oil reservoirs may be the result of mutant resistance. Even if total oil-well nitrate concentrations reach low millimolar levels, the initial concentration of nitrate near the injection site will be much higher than this. For example, the peak nitrate concentration in one study of pulsed nitrate injection was reported as 760 mM (Voordouw et al., 2009). Resistance to nitrate in the presence of a mixed culture is consistent with preliminary fitness profiling data from G20 grown in coculture with the nitrate reducer *Pseudomonas stutzeri* RCH2 in the presence of 100 mM nitrate. Under these mixed-culture conditions, the “nitrate cluster” mutant and the *rex* mutant gained a fitness advantage (A. Deutschbauer, unpublished data) very similar to that observed in pools of G20 mutants alone in the presence of 150 mM nitrate (Table 3).

These fitness studies bring clarity to questions that neither transcriptomic nor proteomic data could answer. While “omics” studies can assist detection and monitoring of changes in the metabolism of bacteria in contaminated environments (Steinberg et al., 2008), they are not sufficient (Torres-García et al., 2009) for elucidating underlying inhibitory mechanisms. In fact, there are poor correlations between the expression of transcripts and the expression of proteins in DvH in response to nitrate stress (Redding et al., 2006; He et al., 2010a). He et al. (2010a) reported 28 genes for which the mRNA and protein levels were both significantly changed in nitrate stress conditions. However, for 7 of these 28 genes, the mRNA was significantly downregulated while the protein was significantly upregulated (He et al., 2010a). Although this poor correlation may be a result of meaningful regulatory mechanisms (Lu et al., 2007), transcript abundance is difficult to interpret and does not always correlate well with gene fitness (Price et al., 2013). For example, there is an upward trend of expression of the “nitrate cluster” genes in both nitrate (He et al., 2010a) and nitrite (He et al., 2006) stress conditions (http://microbesonline.org/). Because mutation of the nitrate cluster genes confers a growth advantage in high nitrate, increased expression of these genes should be detrimental to growth of DvH in high nitrate. This is consistent with the recent deduction (Price et al., 2013) that, counterintuitively, detrimental bacterial genes are often not downregulated. This apparent suboptimal regulation of the nitrate cluster genes in the presence of high concentrations of nitrate likely contributes to the explanation of why their interruption confers such a strong growth advantage.

The roles of the genes of the nitrate cluster in nitrate sensitivity are not immediately obvious from their poor annotation (Table 3). The native functions of the nitrate cluster genes are not likely involved with nitrate, since neither DvH (Seitz and Cypionka, 1986; Pereira et al., 2000) nor G20 (Wall, unpublished data) have been shown to use nitrate for energy conservation. We hypothesize that they may allow non-specific nitrate transport by a leaky thiosulfate transporter. The G20 mutants in this cluster were mildly sick when grown with 10 mM thiosulfate as a terminal electron acceptor (A. Deutschbauer, unpublished data), which suggests a possible role for these genes in thiosulfate uptake. Preliminary data also show that the Δ*rex* DvH strain described here grows more slowly than the parent strain with 30 mM thiosulfate as a terminal electron acceptor (Christensen, unpublished data). Therefore, we suggest that mutation of these genes might relieve nitrate inhibition by barring entry of nitrate into the cell. Further study will be needed to explore their native functions.

Several additional genes outside of the “nitrate cluster” appear to have high fitness values during nitrate stress, indicating that their absence may improve growth. Follow-up studies with individual mutants will be necessary to confirm these predictions. The results from this study clearly indicate that DvH and G20 have common nitrate resistance mechanisms that should be considered in environmental modeling.

## Author contributions

Hannah L. Korte, Samuel R. Fels and Judy D. Wall designed the experiments. Samuel R. Fels conducted the TnLE-seq experiments and analyzed them. Geoff A. Christensen constructed the Δ*rex* mutant and showed its impairment on thiosulfate. Morgan N. Price and Adam M. Deutschbauer generated and analyzed the G20 fitness profiling data. Jennifer V. Kuehl constructed the G20 transposon mutant library. Grant M. Zane constructed the DvH transposon mutant library, confirmed and provided the individual transposon mutants tested. Adam P. Arkin and Judy D. Wall supervised the project. Hannah L. Korte, Morgan N. Price, and Judy D. Wall interpreted the data and wrote the manuscript.

### Conflict of interest statement

The authors declare that the research was conducted in the absence of any commercial or financial relationships that could be construed as a potential conflict of interest.

## References

[B1] American Public Health Association (1992). Standard Methods for The Examination of Water and Waste Water, 18th Edn. Washington, DC: American Water Works Association and Water Pollution Control Federation

[B2] Bender K. S. Yen H. C. B. Hemme C. L. Yang Z. He Z. He Q. (2007). Analysis of a ferric uptake regulator (Fur) mutant of *Desulfovibrio vulgaris* Hildenborough. Appl. Environ. Microbiol. 73, 5389–5400 10.1128/AEM.00276-07 17630305 PMC2042090

[B3] Bradford M. M. (1976). A rapid and sensitive method for the quantitation of microgram quantities of protein utilizing the principle of protein-dye binding. Anal. Biochem. 72, 248–254 10.1016/0003-2697(76)90527-3 942051

[B4] Caffrey S. M. Voordouw G. (2010). Effect of sulfide on growth physiology and gene expression of *Desulfovibrio vulgaris* Hildenborough. Antonie Van Leeuwenhoek 97, 11–20 10.1007/s10482-009-9383-y 19821141

[B5] Callbeck M. C. Agrawal A. Voordouw G. (2013). Acetate production from oil under sulfate-reducing conditions in bioreactors injected with sulfate and nitrate. Appl. Environ. Microbiol. 79, 5059–5068 10.1128/AEM.01251-13 23770914 PMC3754712

[B6] Cataldo D. A. Haroon M. Scharder L. E. Youngs V. L. (1975). Rapid colorimetric determination of nitrate in plant tissue by nitration of salicylic acid. Comm. Soil Sci. Plant Anal. 6, 71–80 10.1080/00103627509366547

[B7] Chardin B. Dolla A. Chaspoul F. Fardeau M. Gallice P. Bruschi M. (2003). Bioremediation of chromate: thermodynamic analysis of the effects of Cr(VI) on sulfate-reducing bacteria. Appl. Microbiol. Biotech. 60, 352–360 10.1007/s00253-002-1091-8 12436319

[B8] Chhabra S. R. He Q. Huang K. H. Gaucher S. P. Alm E. J. He Z. (2006). Global analysis of heat shock response in *Desulfovibrio vulgaris* Hildenborough. J. Bacteriol. 188, 1817–1828 10.1128/JB.188.5.1817-1828.2006 16484192 PMC1426554

[B9] Clark M. E. He Q. He Z. Huang K. H. Alm E. J. Wan X. F. (2006). Temporal transcriptomic analysis as *Desulfovibrio vulgaris* Hildenborough transitions into stationary phase during electron donor depletion. Appl. Environ. Microbiol. 72, 5578–5588 10.1128/AEM.00284-06 16885312 PMC1538716

[B10] Deutschbauer A. Price M. N. Wetmore K. M. Shao W. Baumohl J. K. Xu Z. (2011). Evidence-based annotation of gene function in *Shewanella oneidensis* MR-1 using genome-wide fitness profiling across 121 conditions. PLoS Genet. 7:e1002385 10.1371/journal.pgen.1002385 22125499 PMC3219624

[B11] Elias D. A. Mukhopadhyay A. Joachimiak M. P. Drury E. C. Redding A. M. Yen H. C. B. (2009). Expression profiling of hypothetical genes in *Desulfovibrio vulgaris* leads to improved functional annotation. Nucleic Acids Res. 37, 2926–2939 10.1093/nar/gkp164 19293273 PMC2685097

[B12] Fels S. R. Zane G. M. Blake S. M. Wall J. D. (2013). Rapid Transposon Liquid Enrichment Sequencing (TnLE-seq) for gene fitness evaluation in underdeveloped bacterial systems. Appl. Environ. Microbiol. 79, 7510–7517 10.1128/AEM.02051-13 24077707 PMC3837734

[B13] Gawronski J. D. Wong S. M. S. Giannoukos G. Ward D. V. Akerley B. J. (2009). Tracking insertion mutants within libraries by deep sequencing and a genome-wide screen for *Haemophilus* genes required in the lung. Proc. Natl. Acad. Sci. U.S.A. 106, 16422–16427 10.1073/pnas.0906627106 19805314 PMC2752563

[B14] Green S. J. Prakash O. Jasrotia P. Overholt W. A. Cardenas E. Hubbard D. (2012). Denitrifying bacteria from the genus rhodanobacter dominate bacterial communities in the highly contaminated subsurface of a nuclear legacy waste site. Appl. Environ. Microbiol. 78, 1039–1047 10.1128/AEM.06435-11 22179233 PMC3273022

[B15] Grigoryan A. A. Cornish S. L. Buziak B. Lin S. Cavallaro A. Arensdorf J. J. (2008). Competitive oxidation of volatile fatty acids by sulfate- and nitrate-reducing bacteria from an oil field in Argentina. Appl. Environ. Microbiol. 74, 4324–4335 10.1128/AEM.00419-08 18502934 PMC2493150

[B16] Hauser L. J. Land M. L. Brown S. D. Larimer F. Keller K. L. Rapp-Giles B. J. (2011). Complete genome sequence and updated annotation of *Desulfovibrio alaskensis* G20. J. Bacteriol. 193, 4268–4269 10.1128/JB.05400-11 21685289 PMC3147700

[B17] Haveman S. A. Greene E. A. Stilwell C. P. Voordouw J. K. Voordouw G. (2004). Physiological and gene expression analysis of inhibition of *Desulfovibrio vulgaris* Hildenborough by nitrite. J. Bacteriol. 186, 7944–7950 10.1128/JB.186.23.7944-7950.2004 15547266 PMC529081

[B18] Haveman S. A. Greene E. A. Voordouw G. (2005). Gene expression analysis of the mechanism of inhibition of *Desulfovibrio vulgaris* Hildenborough by nitrate-reducing, sulfide-oxidizing bacteria. Environ. Microbiol. 7, 1461–1465 10.1111/j.1462-2920.2005.00834.x 16104868

[B19] Hazen T. C. Stahl D. A. (2006). Using the stress response to monitor process control: pathways to more effective bioremediation. Curr. Opin. Biotechnol. 17, 285–290 10.1016/j.copbio.2006.03.004 16616486

[B20] He Q. He Z. Joyner D. C. Joachimiak M. Price M. N. Yang Z. K. (2010a). Impact of elevated nitrate on sulfate-reducing bacteria: a comparative study of *Desulfovibrio vulgaris*. ISME J. 4, 1386–1397 10.1038/ismej.2010.59 20445634

[B22] He Q. Huang K. H. He Z. Alm E. J. Fields M. W. Hazen T. C. (2006). Energetic consequences of nitrite stress in *Desulfovibrio vulgaris* Hildenborough, inferred from global transcriptional analysis. Appl. Environ. Microbiol. 72, 4370–4381 10.1128/AEM.02609-05 16751553 PMC1489655

[B21] He Z. Zhou A. Baidoo E. He Q. Joachimiak M. P. Benke P. (2010b). Global transcriptional, physiological and metabolite analyses of the responses of *Desulfovibrio vulgaris* Hildenborough to salt adaptation. Appl. Environ. Microbiol. 76, 1574–1586 10.1128/AEM.02141-09 20038696 PMC2832388

[B23] Heidelberg J. F. Seshadri R. Haveman S. A. Hemme C. L. Paulsen I. T. Kolonay J. F. (2004). The genome sequence of the anaerobic, sulfate-reducing bacterium *Desulfovibrio vulgaris* Hildenborough. Nat. Biotech. 22, 554–559 10.1038/nbt959 15077118

[B24] Jalali K. Baldwin S. A. (2000). The role of sulphate reducing bacteria in copper removal from aqueous sulphate solutions. Water Res. 34, 797–806 10.1016/S0043-1354(99)00194-3

[B25] Jiang W. Fan W. (2008). Bioremediation of heavy metal-contaminated soils by sulfate-reducing bacteria. Ann. N.Y. Acad. Sci. 1140, 446–454 10.1196/annals.1454.050 18991946

[B26] Keller K. L. Bender K. S. Wall J. D. (2009). Development of a markerless genetic exchange system for *Desulfovibrio vulgaris* Hildenborough and its use in generating a strain with increased transformation efficiency. Appl. Environ. Microbiol. 75, 7682–7691 10.1128/AEM.01839-09 19837844 PMC2794091

[B27] Keller K. L. Rapp-Giles B. J. Semkiw E. S. Porat I. Brown S. D. Wall J. D. (2014). New model for electron flow for sulfate reduction in Desulfovibrio alaskensis G20. Appl. Environ. Microbiol. 80, 855–868 10.1128/AEM.02963-13 24242254 PMC3911205

[B28] Keller K. L. Wall J. D. Chhabra S. (2011). Methods for engineering sulfate reducing bacteria of the genus *Desulfovibrio*. Methods Enzymol. 497, 503–517 10.1016/B978-0-12-385075-1.00022-6 21601101

[B29] Kircher M. Sawyer S. Meyer M. (2012). Double indexing overcomes inaccuracies in multiplex sequencing on the Illumina platform. Nucleic Acids Res. 40:e3 10.1093/nar/gkr771 22021376 PMC3245947

[B30] Langridge G. C. Phan M. D. Turner D. J. Perkins T. T. Parts L. Haase J. (2009). Simultaneous assay of every *Salmonella typhi* gene using one million transposon mutants. Genome Res. 19, 2308–2316 10.1101/gr.097097.109 19826075 PMC2792183

[B31] Larsen R. A. Wilson M. M. Guss A. M. Metcalf W. W. (2002). Genetic analysis of pigment biosynthesis in *Xanthobacter autotrophicus* Py2 using a new, highly efficient transposon mutagenesis system that is functional in a wide variety of bacteria. Arch. Microbiol. 178, 193–201 10.1007/s00203-002-0442-2 12189420

[B32] Li M. Z. Elledge S. J. (2007). Harnessing homologous recombination *in vitro* to generate recombinant DNA via SLIC. Nat. Methods 4, 251–256 10.1038/nmeth1010 17293868

[B33] Ligthelm D. J. De Boer R. B. Brint J. F. Schulte W. M. (1991). Reservoir souring. An analytical model for H2S generation and transportation in an oil reservoir owing to bacterial activity, in Offshore Europe 91—Proceedings, (Aberdeen, Scotland: Society of Petroleum Engineers of AIME), 369–378 10.2118/23141-MS

[B34] Lloyd J. R. Ridley J. Khizniak T. Lyalikova N. N. Macaskie L. E. (1999). Reduction of technetium by *Desulfovibrio desulfuricans*: biocatalyst characterization and use in a flowthrough bioreactor. Appl. Environ. Microbiol. 65, 2691–2696 10347062 10.1128/aem.65.6.2691-2696.1999 PMC91397

[B35] Lovley D. R. Roden E. E. Phillips E. J. P. Woodward J. C. (1993a). Enzymatic iron and uranium reduction by sulfate-reducing bacteria. Marine Geol. 113, 41–53 10.1016/0025-3227(93)90148-O23373896

[B36] Lovley D. R. Widman P. K. Woodward J. C. Phillips E. J. P. (1993b). Reduction of uranium by cytochrome c_3_ of *Desulfovibrio vulgaris*. Appl. Environ. Microbiol. 59, 3572–3576 8285665 10.1128/aem.59.11.3572-3576.1993 PMC182500

[B37] Lu P. Vogel C. Wang R. Yao X. Marcotte E. M. (2007). Absolute protein expression profiling estimates the relative contributions of transcriptional and translational regulation. Nat. Biotech. 25, 117–124 10.1038/nbt1270 17187058

[B38] Martins M. Faleiro M. L. Barros R. J. Veríssimo A. R. Barreiros M. A. Costa M. C. (2009). Characterization and activity studies of highly heavy metal resistant sulphate-reducing bacteria to be used in acid mine drainage decontamination. J. Hazard. Mater. 166, 706–713 10.1016/j.jhazmat.2008.11.088 19135795

[B39] McCready R. G. L. Gould W. D. Cook F. D. (1983). Respiratory nitrate reduction by *Desulfovibrio* sp. Arch. Microbiol. 135, 182–185 10.1007/BF00414476

[B40] Mukhopadhyay A. He Z. Alm E. J. Arkin A. P. Baidoo E. E. Borglin S. C. (2006). Salt stress in *Desulfovibrio vulgaris* Hildenborough: an integrated genomics approach. J. Bacteriol. 188, 4068–4078 10.1128/JB.01921-05 16707698 PMC1482918

[B41] Mukhopadhyay A. Redding A. M. Joachimiak M. P. Arkin A. P. Borglin S. E. Dehal P. S. (2007). Cell-wide responses to low-oxygen exposure in *Desulfovibrio vulgaris* Hildenborough. J. Bacteriol. 189, 5996–6010 10.1128/JB.00368-07 17545284 PMC1952033

[B42] Oh J. Fung E. Price M. N. Dehal P. S. Davis R. W. Giaever G. (2010). A universal tagmodule collection for parallel genetic analysis of microorganisms. Nucleic Acids Res. 38, 14 10.1093/nar/gkq419 20494978 PMC2919733

[B43] Parks J. M. Johs A. Podar M. Bridou R. Hurt R. A. Jr. Smith S. D. (2013). The genetic basis for bacterial mercury methylation. Science 339, 1332–1335 10.1126/science.1230667 23393089

[B44] Pereira I. A. LeGall J. Xavier A. V. Teixeira M. (2000). Characterization of a heme c nitrite reductase from a non-ammonifying microorganism, *Desulfovibrio vulgaris* Hildenborough. Biochim. Biophys. Acta 1481, 119–130 10.1016/S0167-4838(00)00111-4 11004582

[B45] Pierce S. E. Davis R. W. Nislow C. Giaever G. (2007). Genome-wide analysis of barcoded *Saccharomyces cerevisiae* gene-deletion mutants in pooled cultures. Nat. Protoc. 2, 2958–2974 10.1038/nprot.2007.427 18007632

[B46] Pierce S. E. Fung E. L. Jaramillo D. F. Chu A. M. Davis R. W. Nislow C. (2006). A unique and universal molecular barcode array. Nat. Methods 3, 601–603 10.1038/nmeth905 16862133

[B47] Postgate J. R. (1984). The Sulfate-Reducing Bacteria, 2nd Edn. Cambridge: Cambridge University Press

[B48] Price M. N. Deutschbauer A. M. Skerker J. M. Wetmore K. M. Ruths T. Mar J. S. (2013). Indirect and suboptimal control of gene expression is widespread in bacteria. Mol. Syst. Biol. 9, 660 10.1038/msb.2013.16 23591776 PMC3658271

[B49] Ravcheev D. A. Li X. Latif H. Zengler K. Leyn S. A. Korostelev Y. D. (2012). Transcriptional regulation of central carbon and energy metabolism in bacteria by redox-responsive repressor rex. J. Bacteriol. 194, 1145–1157 10.1128/JB.06412-11 22210771 PMC3294762

[B50] Redding A. M. Mukhopadhyay A. Joyner D. C. Hazen T. C. Keasling J. D. (2006). Study of nitrate stress in *Desulfovibrio vulgaris* Hildenborough using iTRAQ proteomics. Brief. Funct. Genomic. Proteomic. 5, 133–143 10.1093/bfgp/ell025 16772278

[B51] Riley R. Zachara J. (1992). Chemical Contaminants on DOE Lands and Selection of Contaminant Mixtures for Subsurface Science Research DOE/ER-0547T. Washington, DC: US Department of Energy

[B52] Seitz H. J. Cypionka H. (1986). Chemolithotrophic growth of *Desulfovibrio desulfuricans* with hydrogen coupled to ammonification of nitrate or nitrite. Arch. Microbiol. 146, 63–67 10.1007/BF00690160

[B53] Steinberg C. E. W. Stürzenbaum S. R. Menzel R. (2008). Genes and environment - striking the fine balance between sophisticated biomonitoring and true functional environmental genomics. Sci. Total Environ. 400, 142–161 10.1016/j.scitotenv.2008.07.023 18817948

[B54] Sunde E. Thorstenson T. Torsvik T. Vaag J. E. Espedal M. S. (1993). Field-related mathematical model to predict and reduce reservoir souring, in Proceedings of the 1993 SPE International Symposium on Oilfield Chemistry, (Society of Petroleum Engineers, Inc.), 449–456

[B55] Sunde E. Torsvik T. (2005). Microbial control of hydrogen sulfide production in oil resevoirs, in Petroleum Microbiology, eds Ollivier B. Magot M. (Washington, DC: ASM Press), 201–213

[B56] Torres-García W. Zhang W. Runger G. C. Johnson R. H. Meldrum D. R. (2009). Integrative analysis of transcriptomic and proteomic data of *Desulfovibrio vulgaris*: a non-linear model to predict abundance of undetected proteins. Bioinformatics 25, 1905–1914 10.1093/bioinformatics/btp325 19447782 PMC2712339

[B57] Van Opijnen T. Bodi K. L. Camilli A. (2009). Tn-seq: high-throughput parallel sequencing for fitness and genetic interaction studies in microorganisms. Nat. Methods 6, 767–772 10.1038/nmeth.1377 19767758 PMC2957483

[B58] Voordouw G. Grigoryan A. A. Lambo A. Lin S. Park H. S. Jack T. R. (2009). Sulfide remediation by pulsed injection of nitrate into a low temperature Canadian heavy oil reservoir. Environ. Sci. Technol. 43, 9512–9518 10.1021/es902211j 20000549

[B59] Walian P. J. Allen S. Shatsky M. Zeng L. Szakal E. D. Liu H. (2012). High-throughput Isolation and characterization of untagged membrane protein complexes: outer membrane complexes of *Desulfovibrio vulgaris*. J. Proteome Res. 11, 5720–5735 10.1021/pr300548d 23098413 PMC3516867

[B60] Wall J. Bill Yen H. C. Drury E. C. (2007). Evaluation of stress response in sulphate-reducing bacteria through genome analysis, in Sulphate-Reducing Bacteria: Environmental and Engineered Systems, eds Barton L. L. Hamilton W. A. (New York, NY: Cambridge University Press), 141–165 10.1017/CBO9780511541490.005

[B61] Wall J. D. Rapp-Giles B. J. Rousset M. (1993). Characterization of a small plasmid from Desulfovibrio desulfuricans and its use for shuttle vector construction. J. Bacteriol. 175, 4121–4128 8320227 10.1128/jb.175.13.4121-4128.1993 PMC204841

[B62] Zane G. M. Bill Yen H. C. Wall J. D. (2010). Effect of the deletion of *qmoABC* and the promoter-distal gene encoding a hypothetical protein on sulfate reduction in *Desulfovibrio vulgaris* Hildenborough. Appl. Environ. Microbiol. 76, 5500–5509 10.1128/AEM.00691-10 20581180 PMC2918943

