# Supplementary material for: Genetic basis for nitrate resistance in Desulfovibrio strains
Source: Front Microbiol. 2014 Apr 21;5:153. doi: 10.3389/fmicb.2014.00153 (PMC4001038; doi:10.3389/fmicb.2014.00153)
Supplement: Supplementary file 1 [file DataSheet1.PDF]

Table S1. Quality metrics for *D. alaskensis* G20 fitness profiling experiments

Experiment	Strain Correlation[a]	Operon Correlation[b]
No stress Control	0.650	0.416
150 mM Sodium Nitrate	0.311	0.377
150 mM Potassium Nitrate	0.317	0.393
150 mM Sodium Chloride	0.843	0.490
150 mM Potassium Chloride	0.907	0.493
0.25 mM Sodium Nitrite	0.659	0.409

[a]The correlation of the strain fitness values for the strains present in both pools: 1091 strains measured

[b]The correlation of gene fitness values between adjacent genes predicted (http://www.microbesonline.org/) to be in the same operon

Table S2. Primers used for PCR amplification, Southern probe generation and sequencing in *D. vulgaris*

Primer name	Primer sequence (5’-3’)	Application
DVU0916-1	GCCTTTTGCTGGCCTTTTGCTCACATGATGCTGAGAAGTTCGGTCCGAAG	For amplification of DVU0916 upstream region from gDNA with DVU0916-2 primer to make pMO3311. Underlined portion used as overhang for SLIC with Sp^r,pUC *ori* fragment (SpecRpUC-R). Amplification of Southern probe for confirmation of DVU0916 deletion. forward
DVU0916-2	GCGACAAGATATTCGGCACCAAGTAAGCGTTCGTTAACTTCACTTTTTGCAATGCAC	For amplification of DVU0916 upstream from gDNA with DVU0916-1 primer to make pMO3311. Underlined portion used as overhang for SLIC with Km^r, *upp* fragment (UppCterm). Amplification of Southern probe for confirmation of DVU0916 deletion. reverse
DVU0916-3	GCGCCCCAGCTGGCAATTCCGGCTGGAGCGTGAACGCCTCC	For amplification of DVU0916 downstream from gDNA with DVU0916-4 to make pMO3311. Underlined portion used as overhang for SLIC with Km^r, *upp* fragment (KanPromNterm). forward
DVU0916-4	GTCGAGGCATTTCTGTCCTGGCTGGGATTTCATGGGCCCCGATGTATTGG	For amplification of DVU0916 upstream region from gDNA with DVU0916-3 primer to make pMO3311. Underlined portion used as overhang for SLIC with Sp^r,pUC *ori* fragment (SpecRpUC-F). reverse
SpecRpUC-F	CCAGCCAGGACAGAAATGCCTCG	For amplification of Sp^r and pUC *ori* from pMO719 to make pMO3311. Used as overhang for SLIC. forward
SpecRpUC-R	ATGTGAGCAAAAGGCCAGCAAAAGGC	For amplification of Sp^r and pUC *ori* from pMO719 to make pMO3311. Used as overhang for SLIC. reverse
KanPromNterm	CCGGAATTGCCAGCTGGG	For amplification of Km^r from pMO719 to to make pMO3311. Used as overhang for SLIC. forward
UppCTerm	CTTACTTGGTGCCGAATATCTTGTCGC	For amplification of Km^r from pMO719 to to make pMO3311. Used as overhang for SLIC. reverse
SpecRpUC-up	GGGAAACGCCTGGTATCTTTATAGTCCT	For colony PCR, screen of and sequencing upstream region of pMO3311 deletion cassette. forward
pMO719XbaI-Dn	TGGGTTCGTGCCTTCATCCG	For colony PCR, screen of and sequencing downstream region of pMO3311 deletion cassette; also for sequencing complementation constructs. Sequencing primer to confirm inserts into pMO9075 for complementation of DVU0916 (pMO3313). reverse
Kan-int-Fwd-rev-comp	CTCATCCTGTCTCTTGATCAGATCT	For sequencing downstream region of pMO3311 deletion cassette. forward
DvH-Upp gene Cterm-out	GCTGAAGCGCATCGTGGACAA	For sequencing upstream region of pMO3311 deletion cassette. reverse
pBG1-2199-F	GCTGAAAGCGAGA	Sequencing primer to confirm inserts into pMO9075 for

	AGAGCGCAC	complementation of DVU0916 (pMO3313).
DVU0916-UP-int-F	CCTACGGCCAACGTCAACACCAAC	Sequencing primer to confirm upstream region of deletion cassette of pMO3311. forward
DVU0916-UP-int-R	GTTGGTGTTGACGTTGGCCGTAGG	Sequencing primer to confirm upstream region of deletion cassette of pMO3311. reverse
DVU0916-DWN-int-F	GGATAGCGTGACATTCCCGGACGTG	Sequencing primer to confirm downstream region of deletion cassette of pMO3311. forward
DVU0916-DWN-int-R	CACGTCCGGGAATGTCACGCTATCC	Sequencing primer to confirm downstream region of deletion cassette of pMO3311. reverse
SLIC-DVU0916-comp-F	AGGTTGGGAAGCCCTGCAATGCAGTCCCAGGAGGTACCATATGACCAACATCAAAAGCGAACACATCC	For amplification of DVU0916 to make pMO3313 complementation construct. Underlined portion used as overhang for SLIC assembly with pMO9075 fragment. forward
SLIC-DVU0916-comp-R	GATCGTGATCCCCTGCGCCATCAGATCCTTGCTATTTGTTGCGCGAGAACGTGATGTT	For amplification of DVU0916 to make pMO3313 complementation construct. Underlined portion used as overhang for SLIC assembly with pMO9075 fragment. reverse
pMO9075-SLIC-F	CAAGGATCTGATGGCGCAGGG	For amplification of pMO9075 fragment for SLIC to make pMO3313 complementation construct. forward
pMO9075-SLIC-R3	CTGGGACTGCATTGCAGGGCTTCCCAACCT	For amplification of pMO9075 fragment for SLIC to make pMO3313 complementation construct. reverse

Table S3. ***D. alaskensis*** **G20 fitness profiling results in lactate-sulfate medium with top ten fitness scores in sodium nitrate compared with other amendments**

		No Stress		150 mM Sodium Nitrate		150 mM Potassium Nitrate		150 mM Sodium Chloride		150 mM Potassium Chloride		0.25 mM Sodium Nitrite	
Gene (Dde)	Annotation	Mean Log_2 Ratio[a]	Z Score	**Mean Log_2 Ratio**	Z Score	**Mean Log_2 Ratio**	Z Score	Mean Log_2 Ratio	Z Score	Mean Log_2 Ratio	Z Score	Mean Log_2 Ratio	Z Score
2702	Rex, DNA-binding protein	-2.05	-3.07	**4.23**	3.33	**4.51**	3.42	-1.57	-3.63	-1.43	-3.88	-1.63	-3.05
0601	phosphate/ phosphonate transport system, putative	-0.04	-0.11	**3.51**	3.86	**3.53**	3.66	-0.10	-1.39	-0.05	-1.15	0.06	0.42
0604	PEP/pyruvate binding domain protein	-0.11	-1.08	**3.41**	3.80	**3.42**	3.80	0.01	0.20	0.07	0.81	-0.02	-0.04
0603	RR receiver domain-containing	0.12	0.41	**3.08**	2.84	**3.10**	3.12	0.06	0.95	0.03	0.37	0.08	0.46
0598	DUF	0.12	1.04	**3.01**	3.49	**3.03**	3.80	0.07	0.88	0.01	0.16	0.03	0.35
0600	CHyp	-0.11	-0.52	**2.88**	2.91	**2.87**	2.90	0.05	0.45	-0.01	-0.09	-0.17	-0.91
1268	Na+/proline symporter	-0.01	-0.04	**2.42**	2.71	**2.36**	2.67	0.14	1.05	0.05	0.65	0.24	1.24
0602	Sensory box sensor HK/RR, putative	0.21	0.92	**2.34**	1.91	**2.94**	2.66	0.07	0.88	0.08	1.03	0.03	0.29
0597	DUF	0.07	0.41	**2.25**	3.76	**2.38**	3.76	-0.03	-0.28	0.00	0.12	0.05	0.68
0605	Dual specificity phosphatase, catalytic domain	0.03	0.31	**2.08**	2.79	**2.23**	2.98	0.03	0.55	0.00	0.06	0.01	0.15

[a] Mean Log_2 Ratio = Gene Fitness Score = average of individual mutant fitness scores for mutations of that gene.

Individual mutant fitness score = $\log_2\left(\frac{\text{barcode microarray signal from target mutant cells after stress}}{\text{barcode microarray signal from target mutant cells prior to stress}}\right)$. Signals used to calculate fitness scores were determined by microarray hybridization of barcode tags and normalized as described in the methods.

Table S4. Selected results of fitness profiling of *D. vulgaris* Hildenborough--top ten fitness scores in sodium nitrate compared with MOLS4

**Fitness**	**Gene annotation in *D. vulgaris***	fitness score[a]	fitness score
gene		MOLS4	MOLS4 + 100 mM NO_3
DVU0251	membrane protein, putative (TIGR)	2.05[b]	11.44
DVU0247	response regulator (TIGR), ntrX	1.23[b]	9.14
DVUA0023	ABC transporter, permease protein, putative (TIGR), atoC	0.18	6.10
DVU0249	conserved hypothetical protein (TIGR)	0.26	3.86
DVU0916	AT-rich DNA-binding protein (TIGR)	-2.86	3.81
DVU0123	membrane protein, putative (TIGR)	0.80	3.01
DVU2515	HD domain protein (TIGR)	-0.09	2.95
DVU0540	sensor histidine kinase (TIGR)	0.75	2.77
DVU1999	sulfate transporter family protein (TIGR)	-0.80	2.20
DVU0246	pyruvate phosphate dikinase, PEP/pyruvate binding domain protein (TIGR)	0.36	2.18

$$^{a}\text{Fitness score of a DvH gene} = \log_2 \left[\left(\frac{\#\ \text{insertions in gene}}{\text{length of gene}} \right) / \left(\frac{\#\ \text{insertions in } \textbf{all} \text{ genes}}{\text{length of } \textbf{all} \text{ genes}} \right) \right]$$

[b]These values are artificially inflated because of "barcode bleed" (Kircher et al., 2012), an artifact of having both of these pools on the same Illumina[TM] HiSeq lane (Fels et al., 2013). A similar analysis of WT *D. vulgaris* in MOYLS4 (MOLS4 with 0.1% wt/vol yeast extract), unaffected by barcode bleed, resulted in fitness of 1.10 for DVU0251 and 1.75 for DVU0247 (Fels et al., 2013). As these genes are not predicted to be involved in amino acid biosynthesis, it is expected that their fitness values in MOLS4 should be similar to this original MOYLS4 fitness experiment.
